# Altered Coupling of Cerebral Blood Flow and Functional Connectivity Strength in First-Episode Schizophrenia Patients With Auditory Verbal Hallucinations

**DOI:** 10.3389/fnins.2022.821078

**Published:** 2022-04-25

**Authors:** Jingli Chen, Kangkang Xue, Meng Yang, Kefan Wang, Yinhuan Xu, Baohong Wen, Jingliang Cheng, Shaoqiang Han, Yarui Wei

**Affiliations:** Department of Magnetic Resonance Imaging, The First Affiliated Hospital of Zhengzhou University, Zhengzhou, China

**Keywords:** cerebral blood flow, functional connectivity, neurovascular coupling, auditory verbal hallucination, forward model

## Abstract

**Objective:**

Auditory verbal hallucinations (AVHs) are a major symptom of schizophrenia and are connected with impairments in auditory and speech-related networks. In schizophrenia with AVHs, alterations in resting-state cerebral blood flow (CBF) and functional connectivity have been described. However, the neurovascular coupling alterations specific to first-episode drug-naïve schizophrenia (FES) patients with AVHs remain unknown.

**Methods:**

Resting-state functional MRI and arterial spin labeling (ASL) was performed on 46 first-episode drug-naïve schizophrenia (FES) patients with AVHs (AVH), 39 FES drug-naïve schizophrenia patients without AVHs (NAVH), and 48 healthy controls (HC). Then we compared the correlation between the CBF and functional connection strength (FCS) of the entire gray matter between the three groups, as well as the CBF/FCS ratio of each voxel. Correlation analyses were performed on significant results between schizophrenia patients and clinical measures scale.

**Results:**

The CBF/FCS ratio was reduced in the cognitive and emotional brain regions in both the AVH and NAVH groups, primarily in the crus I/II, vermis VI/VII, and cerebellum VI. In the AVH group compared with the HC group, the CBF/FCS ratio was higher in auditory perception and language-processing areas, primarily the left superior and middle temporal gyrus (STG/MTG). The CBF/FCS ratio in the left STG and left MTG positively correlates with the score of the Auditory Hallucination Rating Scale in AVH patients.

**Conclusion:**

These findings point to the difference in neurovascular coupling failure between AVH and NAVH patients. The dysfunction of the forward model based on the predictive and computing role of the cerebellum may increase the excitability in the auditory cortex, which may help to understand the neuropathological mechanism of AVHs.

## Introduction

Auditory verbal hallucinations (AVHs) are cardinal symptoms in schizophrenia. Defined as the auditory experience of “hearing voices” in the absence of external stimuli that cause them, AVH is suffered by 60–80% of the patients and often produces distress, functional disability, and behavioral alterations (Jardri et al., 2011; Hjelmervik et al., 2020). Considering the severe cognitive problems, poor quality of life, and high morbidity, the physiological mechanism underlying AVHs should be fully understood to promote effective treatment.

Many models have been proposed to account for the different mechanisms of AVHs involving a wide range of brain regions far beyond the auditory cortex in schizophrenia, including into the thalamus (Ferri et al., 2018) and cerebellum (Pinheiro et al., 2020a). Especially, in recent decades, neuroimaging techniques have provided evidence for the central role of the cerebellar circuit in the forward model, which links AVH patients to impaired cerebellar function or structure by erratic prediction and imprecise computation of sensory consequences and also affects higher-level cognitive processes (Sokolov et al., 2017; Moberget and Ivry, 2019; Pinheiro et al., 2020b). Predictive timing disturbances in the forward model are a special marker of SZ and have been associated with other cognitive dysfunctions documented in prior studies (Ciullo et al., 2018). The forward model suggests that the cerebellum compares expected and actual sensory feedback (Martha et al., 2018). Sensory error messages are specifically encoded in the cerebellum’s Purkinje cells’ complicated spike discharges (Brooks et al., 2015). The cerebellar output is small if the entering stimulus matches the predicted one; if a discrepancy–error message is received, activity in the cerebellum increases, and a vast area of cerebral cortex is alerted by increasing excitability (Marco Molinari et al., 2008). In schizophrenia patients with AVHs, differences are sent to cortical regions such as the left superior and middle temporal gyrus (STG/MTG) via the thalamus (Tourville et al., 2008). One previous study reported that an acute brain disorder causes interruption of the excitatory projections from the lesioned brain area to the anatomically intact brain regions (Warren et al., 1958). Similar to the former, a unilateral cerebellar lesion decreased the contralateral cortical excitability and remained a baseline hemispheric CBF unchanging contralateral to a cerebellar lesion, which was suggested to have impaired neurovascular coupling between the cerebellum and cerebral cortex (Enager et al., 2004). Furthermore, a corticocerebellar–thalamic–cortical circuit connects the cerebellum to numerous areas of the cerebral cortex, and the cerebellum may play a key role in this circuit in psychosis by coordinating or modulating elements of cortical activity (Andreasen and Pierson, 2008; Cao et al., 2018). If this circuitry is disrupted, it will cause “cognitive dysmetria,” which is difficult to prioritize, process, coordinate, and respond to information, eventually leading to function decoupling (Nancy et al., 1998). However, how the neurovascular coupling alteration involved in information processing in these brain regions within these circuits is disrupted remains unknown.

In recent years, to get a deeper understanding of the alteration of neurovascular relationships in neurological diseases, researchers began to use the method of neurovascular coupling to explore the pathogenesis of diseases, such as primary open-angle glaucoma (Wang Q. et al., 2021), bipolar disorder and major depressive disorder (He Z. et al., 2019), and Alzheimer’s disease (Drzezga et al., 2011). The method of neurovascular coupling, reflected by cerebral blood flow (CBF), functional connectivity strength (FCS), and their relationship, showed a direct relationship among functional activity, metabolism, and neural activity, which demonstrated that brain regions with higher spontaneous neural activity tend to have more robust connectivity and increased perfusion (Liang et al., 2013). Regional CBF is tightly coupled with brain metabolism and can be measured utilizing functional neuroimaging techniques, such as arterial spin labeling (ASL), which has been widely used in schizophrenia (Vaishnavi et al., 2010; Zhuo et al., 2017; Jing et al., 2018). Increased CBF of the left STG was found in AVH patients accompanied by the left MTG by using this technique (Homan et al., 2012; Zhu et al., 2017; Zhuo et al., 2017). The whole-brain functional connectivity strength (FCS) highlights the involvement of each voxel in transmitting information in the overall brain network by depicting a specific voxel as well as all other voxels in the brain that surpassed a predetermined optimum threshold (Liang et al., 2013; van Lutterveld et al., 2014; Li et al., 2020). There are functional connectivity measures on how well a local activity is integrated across brain regions, which help researchers better understand the dysfunctions in integrated brain networks and the exact coordination of inter-regions in schizophrenia (Lui et al., 2010). In the graph theory, the FCS is also known as “degree centrality” of weighted networks (Wang Y. et al., 2021), and brain areas with a high FCS are regarded functional hubs that are well connected to the rest of the brain (Liang et al., 2013). The whole-brain functional connectivity approach solves some of the limitations of seed-based rsFC analysis and independent component analysis (ICA), all of which are approaches in quantifying rsFC alterations. For example, in the absence of the underlying pathophysiology for a disease, the analysis of seed-based rsFC approach may be difficult due to the requirement for *a priori* definition of seed regions (Nair et al., 2014). The ICA may face uncertainty about the optimal number of components, contentious criteria for discriminating between noise and signal, and interpretive complications brought by a sophisticated algorithm (Fox and Raichle, 2007). Increased FCS in the left crus I, bilateral crus II, left cerebellum VI, vermis VI, vermis VII, and decreased FCS in the left temporal cortex have been discovered using the FCS approach to explore connection alterations in schizophrenia (Wang et al., 2017; Zhu et al., 2017; Basavaraju et al., 2019; Ding et al., 2019).

The CBF–FCS correlation measures the spatial consistency of CBF and FCS across voxels over the entire gray matter (Zhu et al., 2017). The metabolic demand per unit of connection strength is measured by the CBF/FCS ratio, which indirectly indicates the neurovascular coupling of a single voxel or local region (Zhu et al., 2017). Cerebral volume reduction, neural loss, abnormal astrocytes, and white matter pathway interruptions that may contribute to the neurovascular decoupling have been reported in recent studies (Cocchi et al., 2014; Wang Q. et al., 2021). Voxel-wise entire brain studies of the CBF/FCS ratios and CBF–FCS correlations can provide more precise and sensitive information on the alterations in brain functional regions than voxel-wise whole-brain analysis of CBF and FCS indices simply. Using the approach of neurovascular coupling in individuals with schizophrenia, Zhu et al. discovered reduced CBF/FCS ratios in higher-order brain systems related to cognitive control and affective regulation and elevated CBF/FCS ratios in lower-order brain systems, such as sensory processing (Zhu et al., 2017). Unfortunately, up until now, no study has investigated the alteration in neurovascular coupling specific to first-episode drug-naïve schizophrenia (FES) patients with AVHs (AVH).

**FIGURE 1:**
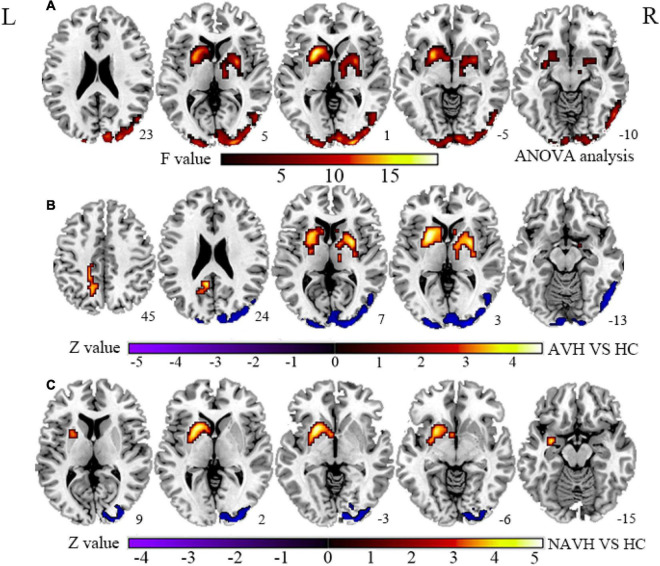
**(A)** Brain regions with significant CBF changes between the AVH, NAVH, and HC groups. **(B)** Brain regions with significant CBF changes in the AVH group. **(C)** Brain regions with significant CBF changes in the NAVH group. CBF, cerebral blood flow; AVH, schizophrenia patients with AVHs; NAVH, schizophrenia patients without AVHs; HC, healthy control.

**FIGURE 2:**
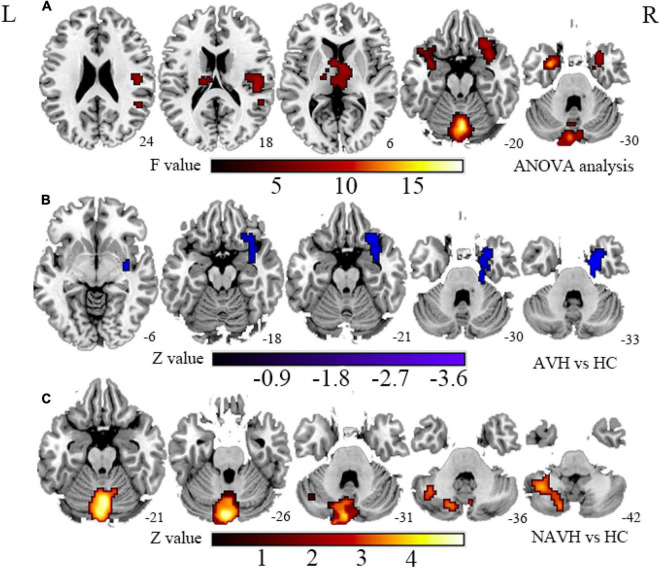
**(A)** Brain regions with significant FCS changes between AVH, NAVH, and HC groups. **(B)** Brain regions with significant FCS changes in the AVH group. **(C)** Brain regions with significant FCS changes in the NAVH group. FCS, functional connectivity strength; AVH, schizophrenia patients with AVHs; NAVH, schizophrenia patients without AVHs.

Considering that patients with AVHs are characterized by impaired information processing related to auditory and speech-related networks in the forward model, which is believed to be associated with common pathological processes of AVHs, we hypothesized that neurovascular coupling alterations should be atypical in AVH patients. Furthermore, because brain regions with CBF and FCS changes are spatially inconsistent, with different effect sizes and directions in the AVH and first-episode drug-naïve schizophrenia (FES) patients without AVHs (NAVH) (Figures 1, 2 and Supplementary Tables 1, 2), we hypothesized that the AVH and NAVH groups would show a reduced and different CBF–FCS coupling, as well as an increased or decreased CBF/FCS ratio accompanied by different CBF and FCS changes. The voxel-based CBF and FCS analyses to detect abnormal perfusion and neural activity in AVH patients and first-episode drug-naïve schizophrenia (FES) patients without AVHs (NAVH) by using ASL and BOLD–fMRI were performed. Three groups were compared on the basis of CBF–FCS coupling in overall gray matter and CBF/FCS ratio voxel-by-voxel.

## Materials and Methods

### Participants

This study recruited 50 FES AVH patients, 50 FES NAVH patients, and 50 age- and sex-matched HC. After preprocessing, we removed zero patients with AVH, five patients with NAVH, and zero patients with HC due to head movement parameters exceeding 3 mm displacement or 3° of rotation. Among the subjects left after preprocessed, we excluded four AVH patients, six NAVH patients, and two HC subjects because they were missing data corresponding to CBF. Finally, included in the study were 46 AVH patients, 39 NAVH patients, and 48 HC. The detailed demographic and clinical data for these participants are shown in Table 1.

**TABLE 1:** Demographic and clinical data of AVH patients, NAVH patients, and HC.

	AVH	NAVH	HC	*F*/*X*^2^/*t-*values	*p-*Values
Number of subjects	46	39	48		
Age (SD)	21.7 (7.86)	20.2 (7.2)	21.9 (7.7)	0.659	0.519
Sex (M/F)	21/25	18/21	23/25	0.033	0.984
Hoffman hallucinations (SD)	23.8 (6.13)	–	–	–	–
**PNASS (SD)**					
Positive	20.1(5.6)	18.1 (6.1)	–	1.474	0.145
Negative	20.2 (5.1)	21.0 (5.8)	–	-0.630	0.531
General	41.6 (7.4)	42.6 (8.6)	–	-0.49	0.626
Total scores	82.3 (14.5)	81.6 (16.4)	–	0.189	0.851
PANSS hallucinations	4.1 (1.6)	2.3 (1.5)		4.125	0.000
PANSS delusions	4.7 (1.4)	3.8 (1.7)		2.107	0.040
FD (SD)	0.12 (0.08)	0.13 (0.10)	0.13 (0.07)	0.369	0.692

*AVH, schizophrenia with AVH patients; NAVH, schizophrenia without AVH patients; HC, healthy control; F, female; M, male; FD, framewise displacement; PNASS, positive and negative syndrome scale.*

The diagnosis of schizophrenia is determined by a psychiatrist and evaluated and confirmed by an experienced psychologist using DSM-5 standards (Vahia, 2013). The Positive and Negative Symptom Scale (PANSS) was used to assess the severity of psychotic symptoms. A total of 46 patients reported experiencing AVHs within the past 4 weeks in the AVH group, most within the past week, while the other 39 patients reported no AVHs in their lifetime or in the past 4 months in the NAVH group. The Auditory Hallucination Rating Scale (AHRS) was used to assess the severity of AVHs. In the end, we collected PANSS data from 46 AVH patients and 39 NAVH patients and AHRS hallucination data from all AVH patients.

For the patient groups, the following are the exclusion criteria: (1) mental disorders caused by physical diseases other than schizophrenia, (2) alcohol addiction or a history of substance abuse, (3) contraindications to MRI, and (4) traumatic head injuries. The exclusion criteria for the HCs are any mental illness, neurological disease, and related family history. The age- and gender-matched HCs were recruited from the same geographic area. Moreover, the same exclusion criteria that were used for SZ patients were used for HCs. All subjects are right-handed. All subjects signed an informed consent form, and this study has been approved by the Ethics Committee of the First Affiliated Hospital of Zhengzhou University.

### Data Acquisition

All subjects who met the enrollment conditions used the same eight-channel 3.0 Tesla magnetic resonance scanner (GE Discovery MR750, United States) to complete the MRI data collection. The collection location was located in the Magnetic Resonance Department of the First Affiliated Hospital of Zhengzhou University. Spatial 3D high-resolution T1-weighted images (3DT1) were acquired using a brain volume sequence with the following settings: repetition time (TR)/echo time (TE) = 8.2/3.2 ms, slice thickness = 1 mm, slice gap = 0 mm, flip angle = 12°, slice number = 1, field of view (FOV) = 25.6 × 25.6 cm^2^, number of averages = 1, matrix size = 256 × 256, and voxel size = 1 × 1 × 1 mm^3^. The resting-state perfusion imaging was performed using a pseudo-continuous ASL (pcASL) sequence with a 3D fast spin–echo acquisition and background suppression, and the parameters are as follows: TR/TE = 4,886/10.5 ms, slice thickness = 4.0 mm, slice gap = 0 mm, flip angle = 111°, slice number = 80, FOV = 24 × 24 cm^2^, number of averages = 3, matrix size = 512 × 512, voxel size = 0.5 × 0.5 × 4 mm^3^. A state of rest BOLD-fMRI data were collected using the following parameters in a gradient-echo single-shot echo-planar imaging (GRE-SS-EPI) sequence: TR/TE = 2,000/30 ms, slice thickness = 4 mm, slice gap = 0.5 mm, flip angle = 90°, slice number = 32, FOV = 22 × 22 cm^2^, number of averages = 1, matrix size = 64 × 64, voxel size = 3.4375 × 3.4375 × 3.4375 mm^3^. The duration of the resting state scan is 6 min.

### fMRI Data Preprocessing

The BOLD-fMRI data are preprocessed by the Data Processing and Analysis of Brain Imaging (DPABI) toolbox^1^, which is based on Statistical Parametric Mapping (SPM12^2^) and MATLAB (MathWorks). The following steps were performed: (1) removing the first five time points; (2) slice timing correction; (3) realigning; if a participant’s maximum head motion was greater than 3 mm or 3° of rotation, they were excluded; (4) normalizing the BOLD-fMRI data space to the template of the Montreal Neurology Institute (resampled voxel size = 3 × 3 × 3 mm^3^); (5) detrending; (6) filtering (0.01–0.08 Hz); (7) scrubbing the BOLD-fMRI data; and (8) regression of the Friston-24 motion parameters, cerebrospinal fluid signal, white matter signal.

### Functional Connectivity Analysis

The FCS of the whole-brain gray matter is the average value of the functional connectivity strength between a given voxel X0 and all other voxels in the whole brain gray matter. Based on the gray matter template provided by the software, the Pearson correlation coefficient between each voxel and other voxel BOLD time series was calculated, the correlation threshold was set at 0.2 (Liu et al., 2015), and the complete gray matter function connection matrix of each subject was obtained. We used an isotropic Gaussian kernel [full width at half maximum (FWHM) = 6 mm] to spatially smooth the FCS map.

### Cerebral Blood Flow Analysis

The CBF images were received from the ASL difference images by subtracting the label images from the control images. The CBF images were processed through a cloud platform (Beijing Intelligent Brain Cloud, Inc.^3^). (1) The CBF images were coregistered to the template of the Montreal Neurology Institute (resampled voxel size = 3 × 3 × 3 mm^3^) and segmented into gray matter, white matter, and cerebrospinal fluid maps. (2) The images were spatially smoothened by using a Gaussian kernel with 6 mm fullwidth at half-maximum (FWHM).

### Cerebral Blood Flow–Functional Connectivity Strength Correlation Analysis

We conducted correlation analyses across voxels for each participant to statistically analyze the correlation relation between CBF and FCS on the entire gray matter. First, the CBF and FCS maps were normalized into Z-scores for each participant by subtracting the mean and dividing by the SD of global values within the gray matter mask. The *df*_*eff*_ of across-voxel correlations was then calculated using the equation below:


$$df_{eff}=\frac{N}{\left(FWHMx\times FWHMy\times FWHMz\right)/v}-2$$

where v is the volume of a voxel (3 × 3 × 3 mm^3^), and N is the number of voxels (*N* = 66,817) used in the analyses. FWHMx × FWHMy × FWHMz were the average smoothness of the CBF and FCS maps (12.1 × 13.0 × 11.9 mm^3^) estimated using Matlab’s DPABI software (DPABI V3.0^4^). In our study, the *df*_*eff*_ of across-voxel correlations was 961. Finally, The CBF–FCS correlation coefficients were compared using one-way ANOVA.

### Cerebral Blood Flow/Functional Connectivity Strength Ratio Analysis

To evaluate the amount of blood supply per unit of connectivity strength, we computed the CBF/FCS ratio of each voxel. Before calculating the CBF/FCS ratio, it is necessary to note that both CBF and FCS are the original values without *Z* transformation. After the ratio, perform *Z* transformation to improve normality. The operation steps are based on Matlab’s DPABI software, using the whole-brain gray matter template as the mask, and calculate the CBF/FCS ratio for each subject.

### Voxel-Wise Comparisons in Cerebral Blood Flow and Functional Connectivity Strength

To further understand what might be causing changes in the CBF/FCS ratio, we analyzed CBF and FCS changes between the three groups voxel-wise while controlling for age, gender, and GMV.

### Clinical and Cognitive Associations

Based on the anatomical template, the average CBF/FCS ratio of the subregions with significant group differences on *F* map were extracted, and the non-parametric Spearman rank correlation analysis (Bonferroni corrected) was used to test the CBF/FCS ratio of each significant subregion and the clinical measures in the AVH and NAVH groups (PANSS positive, negative, general, total score, and AHRS).

### Statistical Analysis

The intergroup differences of voxel-wise CBF, FCS, CBF–FCS correlation, and CBF/FCS ratio were tested by using analysis of variance (ANOVA) with age, gender, and GMV (Crow et al., 1980) of each subject as covariates. Multiple comparisons were corrected according to the Gaussian random field (GRF) theory (voxel-wise *p* < 0.005, cluster-wise *p* < 0.05, two-tailed) in the DPABI toolbox (see text footnote 1)^4^.

### Validation Analysis

The correlation threshold of *r* = 0.2 was applied in the FCS calculation (Liu et al., 2015). We repeatedly computed the whole-brain FCS with correlation thresholds of 0.1 and 0.3 to verify the stability of the results.

## Results

### Spatial Distribution of the Functional Connectivity Strength, Cerebral Blood Flow, and Cerebral Blood Flow/Functional Connectivity Strength Ratio

The geographic distributions of FCS, CBF, and the CBF/FCS ratio were similar in the AVH, NAVH, and HC groups (Supplementary Figure 1). At the level of CBF index, the brain regions of the three groups of HC, AVH, and NAVH that showed similar CBF elevation were distributed in the medial/lateral prefrontal cortex, anterior/posterior cingulate cortex, precuneus, lateral temporal and parietal cortices, sensorimotor, and visual cortices. At the level of FCS index, the brain regions of the three groups of HC, AVH, and NAVH that showed similar FCS elevation were distributed in the lateral temporal cortex, prefrontal cortex, anterior and posterior cingulum, and visual cortex, which were all shown to have higher FCS. At the level of CBF/FCS ratios index, the brain regions of the three groups of HC, AVH, and NAVH that showed similar CBF/FCS ratio elevations were distributed in the medial prefrontal cortex, anterior cingulate cortex, sensorimotor cortex, and thalamus.

### Cerebral Blood Flow–Functional Connectivity Strength Correlation

**FIGURE 3:**
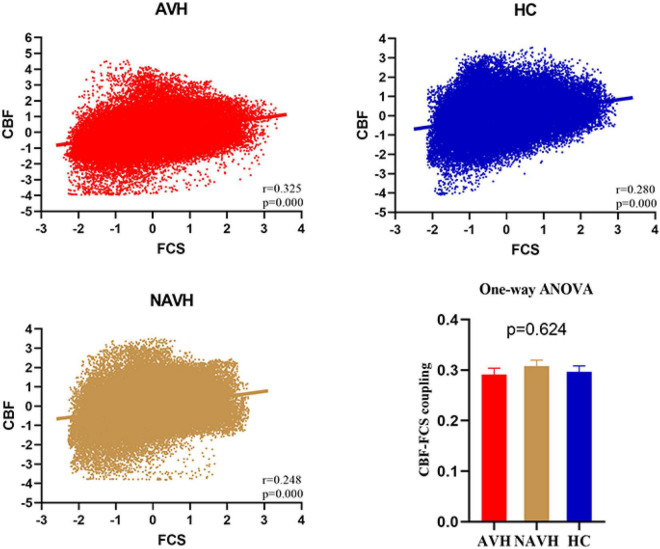
Whole gray matter level cerebral blood flow–functional connectivity strength (CBF–FCS) coupling changes in schizophrenia. Scatter plots of the spatial correlations across voxels between CBF and FCS in an AVH (red), an NAVH patient (yellow), and an HC subject (blue), respectively. The mean whole gray matter level CBF–FCS coupling in AVH patients, NAVH patients, and HC. Although CBF is significantly correlated with FCS in both schizophrenia and control groups, there was no difference between the three groups. CBF, cerebral blood flow; FCS, functional connectivity strength; AVH, schizophrenia patients with AVHs; NAVH, schizophrenia patients without AVHs; HC, healthy control.

Although CBF was significantly correlated with FCS in both AVH, NAVH, and control groups (Figure 3), the three groups had no significant differences in CBF–FCS coupling (one-way ANOVA F = 0.473, *p* = 0.624; Figure 3). *Post-hoc* analysis: AVH group compared with HC (two-sample *t*-test, *t* = -0.343, *p* = 0.732), NAVH compared with HC (two-sample *t*-test, *t* = 0.692, *p* = 0.491), AVH compared with NAVH (two-sample *t*-test, *t* = -1.017, *p* = 0.321).

### Cerebral Blood Flow/Functional Connectivity Strength Ratio

**FIGURE 4:**
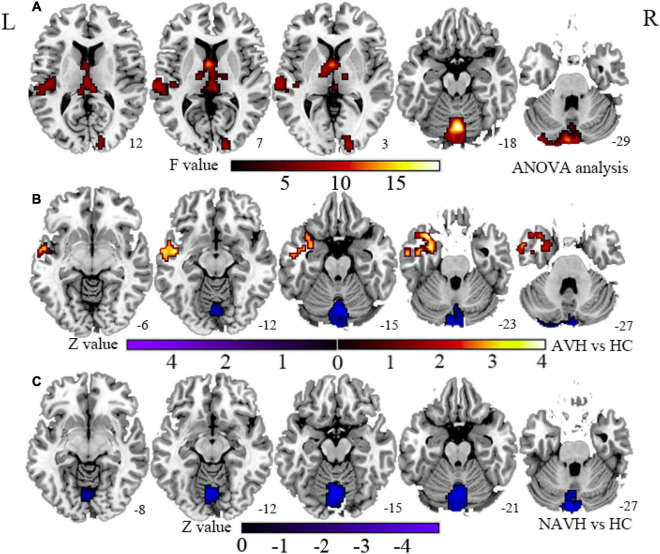
**(A)** CBF/FCS ratio results for HC, AVH, and NAVH groups at a voxel-level height threshold of *p* < 0.005 (two sided) and cluster size GRF corrected threshold of *p* < 0.05. **(B)** Brain regions with significant CBF/FCS changes in the AVH group. **(C)** Brain regions with significant CBF/FCS changes in the NAVH group. CBF, cerebral blood flow; FCS, functional connectivity strength.

Compared with the HC group, both the AVH and NAVH groups exhibited decreased CBF/FCS ratio in left crus I/II, vermis VI/VII, as well as left cerebellum VI. Compared with the HC group, the AVH group showed increased CBF/FCS ratio in the left STG/MTG unparalleled and decreased CBF/FCS ratio in right cerebellum crus II (voxel level *p* < 0.005, cluster level *p* < 0.05, GRF-corrected, Figure 4 and Table 2). Unfortunately, there was no difference in CBF/FCS ratio between the AVH and NAVH groups.

**TABLE 2:** Brain regions with significant group differences in cerebral blood flow/functional connectivity strength (CBF/FCS) ratio.

Group differences	Regions	Cluster size (voxels)	Peak MNI coordinate			Peak *t-*values
			*X*	*Y*	*Z*	
*AVH > HC*	L_STG	98	−63	−24	6	10.91
	L_MTG	35	−45	−21	15	9.35
*AVH/NAVH < HC*	Vermis VI	71	3	−72	−18	19.27
	L_cerebellum crus II	57	−3	−87	−27	−15.00
	L_cerebellum VI	51	−9	−75	−18	−9.10
	L_cerebellum crus I	51	−33	−90	−30	−8.19
	Vermis VII	27	3	−72	−24	−10.30
*AVH < HC*	R_cerebellum crus II	22	6	−87	−27	−10.45

*AVH, schizophrenia with AVHs patients; NAVH, schizophrenia without AVHs patients; HC, healthy control; MNI, Montreal Neurological Institute; L_MTG, left middle temporal gyrus; L_STG, left superior temporal gyrus.*

### Cerebral Blood Flow and Functional Connectivity Strength

Compared with the HC group, the significant brain regions of CBF in AVH and NAVH patients are shown in Figure 1 and Supplementary Table 1 (*p* < 0.05, GRF-corrected), and the FCS is shown in Figure 2 and Supplementary Table 2 (*p* < 0.05, GRF-corrected). The AVH group did not show any difference in CBF compared with the NAVH group (voxel level *p* < 0.005, cluster level *p* < 0.05, GRF-corrected).

### The Correlation Between Cerebral Blood Flow/Functional Connectivity Strength Ratio and Psychotic Symptoms

**FIGURE 5:**
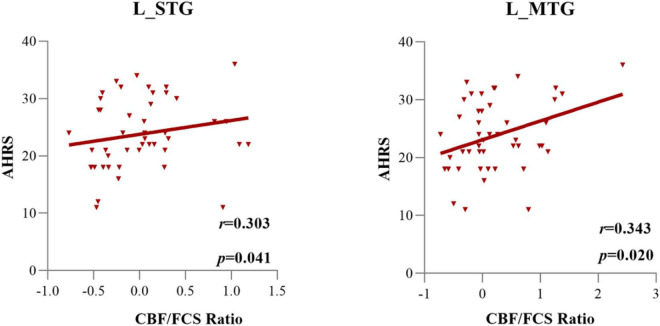
The cerebral blood flow/functional connectivity strength (CBF/FCS) ratio of the left STG and left MTG was positively correlated with the severity of auditory verbal hallucinations, as assessed by AHRS scores in AVH patients.

Supplementary Table 3 shows the associations of the PANSS positive, negative, and general subscores with the normalized CBF/FCS ratio of each significant subregion. Figure 5 and Supplementary Table 4 exhibit the relationships between the CBF/FCS ratio of each meaningful ROI and the AHRS. In the AVH group, we found a significance positive correlation between the CBF/FCS ratio in the left STG/MTG and the AHRS (left MTG: Spearman’s ρ = 0.343, *p* = 0.020; left STG: Spearman’s ρ = 0.303, *p* = 0.041). However, the significance did not survive the Bonferroni correction (*p* < 0.05/51 = 0.001).

### Validation Analysis

We repeated our analysis using correlation thresholds of *r* = 0.1 and *r* = 0.3 to see if the correlation thresholds had any effect on our FCS calculation results. We found that the brain regions with significant CBF/FCS differences at *r* = 0.1 (Supplementary Figure 2 and Supplementary Table 5) were consistent with *r* = 0.2 (Figure 4 and Table 2). With *r* = 0.3 as the threshold (Supplementary Figure 3 and Supplementary Table 6), the vermis VI/VII, left cerebellum crus I/II, left cerebellum VI, and right cerebellum crus II in the AVH and NAVH groups were preserved. The spatial distributions of CBF, FCS, and CBF/FCS ratio at *r* = 0.1 (Supplementary Figure 4) and *r* = 0.3 (Supplementary Figure 5) were very similar to those at *r* = 0.2 (Figure 3). The spatial correlations between CBF and FCS across voxels in anAVH patient (red), an NAVH patient (yellow), and an HC subject (blue) at *r* = 0.1 (Supplementary Figure 6) and *r* = 0.3 (Supplementary Figure 7) were comparable with *r* = 0.2 (Figure 3).

## Discussion

In this study, we adopted the method of neurovascular coupling in three groups (AVH patients, NAVH patients, and HC). Compared with HCs, the two schizophrenia groups showed widespread common decreased CBF/FCS ratios in the cerebellum regions, i.e., the left cerebellar crus I/II, left cerebellum VI, and vermis VI/VII. The AVH group exhibited additional alterations, including increased CBF/FCS ratio in the left STG/MTG and decreased CBF/FCS ratio in the right crus II. These converging results confirmed differences between patients with and without AVHs compared with HCs, respectively, suggesting neural mechanisms for hallucinations.

Increased FCS may reflect a plastic or compensatory response mechanism to structural abnormalities or an undifferentiated state of brain activity characterized by a disruption of usually separated neural activity in schizophrenia (Fornito and Bullmore, 2015). Previous research has found that in schizophrenia, lower structural integrity is accompanied by better functional connectivity, implying that structural impairments can be mitigated by improved functional integration and functional plasticity (Cocchi et al., 2014; Straathof et al., 2018) or excessive attention to internal stimuli (Narr and Leaver, 2015). Cerebellar morphology studies reported reduced GMV in the left cerebellum VI (He H. et al., 2019), left crus I/II (Ding et al., 2019), and cerebellum vermis (Zhong et al., 2018) accompanied by increased FCS between the cerebellum and cortical/subcortical networks or brain regions. Furthermore, structural damage, such as GMV reduction, diminished cortical thickness, and white matter alteration, may impede the exact coordination of inter-regional functional synchronization and reduce information transmission, resulting in decreased FCS in schizophrenia (Kelly et al., 2018; Pan et al., 2019). Previous studies with schizophrenia, for example, have demonstrated a disruption or deviations within the white matter interconnecting left hemisphere language regions (IFG/STG/MTG) accompanied by a low degree of functional connectivity between them (Jeong et al., 2009; de Weijer et al., 2013). The CBF changes in schizophrenia may reflect aberrant neural activity, changes in neurotransmitters, and microvasculature alterations related to neuroinflammation (Kirkpatrick and Miller, 2013; Muller, 2018; Sukumar et al., 2020). Homan et al. has reported that repetitive transcranial magnetic stimulation (TMS) treatment, associated with decreased neural activity, can reduce resting-brain perfusion of the left STG (Homan et al., 2012). AVH patients with persistent regional high perfusion of the left STG indicated that neuronal activity in the left STG was a characteristic biomarker in AVHs (Homan et al., 2013). Groen et al. (2011) showed spontaneous reactivation of memory traces together with increased CBF in the left MTG.

Based on the previous research, any single or a combination of neurovascular unit component impairments and abnormalities (significantly, the astrocytes), neural activity anomaly, or structural impairments can lead to abnormal neurovascular coupling (Howarth, 2014; Stobart and Anderson, 2013), precise coordination and integration of inter-regional functional synchronization is impaired (Pan et al., 2019), and information transmission is decreased eventually (Kelly et al., 2018). Any cause of abnormality in CBF or FCS might lead to a change in the ratio of CBF/FCS. Absolutely, many other factors could also result in neurovascular decoupling, but it is not the focus of our article (Girouard and Iadecola, 2006). The across-voxel correlation between CBF and FCS between the three groups showed no statistical difference on the whole gray matter level in our results. We speculate that the result may be due to the mutual cancelation of many influencing factors as above precisely. However, the CBF/FCS ratio, another coupling index, could show similar discrepancy. The CBF/FCS ratio directly indicated neurovascular coupling of a given voxel or local region and maintained balance in healthy brains (Liang et al., 2013; Zhu et al., 2017). The CBF/FCS ratio equilibrium can be altered in schizophrenia and may differ between AVH and NAVH individuals, with CBF and FCS altering in different directions.

Compared with HC, in the NAVH patients, the left crus I/II, vermis VI/VII, and left cerebellum VI showed increased FCS and normal CBF, suggesting that the decreased CBF/FCS ratio in these regions is predominantly driven by the increased FCS. Compared with HC, in the AVH patients, the decreased CBF/FCS ratio in the left crus I/II, vermis VI/VII, left cerebellum VI, and right crus II was predominantly driven by both CBF and FCS. Under these circumstances, the CBF/FCS ratio decrease was based on ROI analyses in the AVH group (Supplementary Figure 8). Interactions between the posterior lateral cerebellum (e.g., crus I/II) and the prefrontal cortex underlie the engagement of the cerebrum in higher-level functions like cognitive control and processing (Buckner et al., 2011; Carass et al., 2018). Many studies on the cerebellum structure have proven that the reduction in cerebellar gray matter in the left crus I and left crus II was related to the decrease in cognitive function (Kuhn et al., 2012; Kim et al., 2018; Moberget et al., 2018; Uwisengeyimana et al., 2020). In a functional imaging study, the cerebellar VI, which is coupled to subcortical limbic regions and covered in the salience network of the intrinsic connection networks in the cerebral cortex, prioritizes processing of emotionally significant stimuli in a context-dependent way (Seeley et al., 2007; Habas et al., 2009). Moreover, anatomically, the cerebellar vermis VI and VII are located in the posterior section of the cerebellar vermis (Carass et al., 2018), The vermis situated in the midline of the cerebellum is equivalent to the limbic cerebellum, connected to the thalamus and the limbic system (Ichimiya et al., 2001), and plays a role in higher-level functions, such as affection or emotional regulation and cognitive processing (Schmahmann et al., 2007; Yucel et al., 2013; Womer et al., 2016). Therefore, cerebellar VI may have a role in determining the valence of important emotional cues and choosing suitable behavioral responses (Habas et al., 2009). In other words, these regions, which are associated with one of the cerebellum’s major tasks in emotional processing, were activated by computing cues, resulting in diminished consolidation of emotional signals and delivery to the cerebral cortex (Adamaszek et al., 2017). Cerebellum VI is the part of the upper cerebellum that is functionally connected to sensory–motor-related areas. It is also projected to the sensorimotor brain network and is considered to be an important component of motor control and coordination (Grodd et al., 2001; Buckner et al., 2011; Ding et al., 2019). In cognitive processing, the sensorimotor network is involved in perceiving face expressions, emotions, and personal desires (Watanuki et al., 2016). Small cerebellum VI clusters were found in salience and sensorimotor networks. The overlap suggests an intracerebellar connection between them that might relate to limbic control of the motor system (Habas et al., 2009). Therefore, the motor learning of the cerebellum in the forward model may straightly exemplify the intimate relationship between the cerebellum’s motor and cognitive domains (Jacobi et al., 2021).

Pastor et al. discovered that frequency-specific coupling between STG and crus II in the auditory cortical–cerebellar–thalamic loop regulates auditory cortex oscillatory activity in schizophrenia AVHs (Pastor et al., 2008). Furthermore, Coull and Nobre (2008) have demonstrated that cerebellar areas are almost always engaged by explicit time prediction, depending on the individual task setting. Previous research has suggested that the left crus I and bilateral crus II may have a role in AVHs by altering sensory feedback and, as a result, unpredictable prediction, as shown by the forward model (Runnqvist et al., 2016; Pinheiro et al., 2020b). In addition, the posterior vermis has shown involvement in a cerebello–thalamo–cortical circuit for error-related cognitive control in healthy adults (Womer et al., 2016) and auditory prediction in AVHs (Pinheiro et al., 2020a). Given the cerebellum’s role in the forward model and its function as a comparator, the cerebellum can compare actual input with previous stimuli and test for discrepancies. If a discrepancy–error signal is discovered, cerebellar activity increases, and a vast portion of the cerebral cortex is alerted by increasing excitability (Marco Molinari et al., 2008). Our findings of decreased CBF/FCS ratio in cerebellum regions could implicate that cerebellum cognitive dysmetria is linked to gray matter structural damage or disruption of the cerebello-cortical circuit, resulting in patients with AVHs having difficulty synchronizing and integrating neuronal computations and processing to generate ordered and meaningful motor and cognitive activities (Nancy et al., 1998).

Compared with HC, the left STG/MTG showed a significantly increased CBF/FCS ratio in AVH patients. Further analysis based on ROI showed that these areas of AVH patients have higher CBF and lower FCS than HC (Supplementary Figure 8). Many structural (Onitsuka et al., 2004; Cui et al., 2018; Curtis et al., 2021), functional neuroimaging (Allen et al., 2008), and circuit studies (Benetti et al., 2015; Huang et al., 2019; Ren et al., 2021) have indicated that the left STG, particularly the primary and association auditory cortex, and the left MTG, play a key role in the etiology of AVHs. The STG on the left mainly deals with the perception of “speech,” that is, understanding the phonetic and semantic features of the speech content (Modinos et al., 2013) and auditory feedback processing originating from the cerebellum (Christoffels et al., 2007). As we all know, the left MTG is known to be especially vital for the semantic processing of speech and mapping sound to meaning (Clos et al., 2014; Liu et al., 2016). It may be related to the internal attribution of the event. In this case, the self-stored semantic memory is considered an active and intentional agent (Blackwood et al., 2000). Reduced left STG/MTG gray matter volumes are linked to higher AVH severity (Allen et al., 2008). A recent research suggests that AVHs are caused by abnormally high resting-state activity in the auditory cortex (Kuehn and Gallinat, 2012; Hugdahl and Sommer, 2018). AVHs are caused by abnormal height or abnormal static activity in the left STG/MTG, which causes spontaneous internal signals to be misunderstood as external (Cho and Wu, 2013; Alderson-Day et al., 2015). Internal speech being mistakenly attributed to external or non-self sources could be the result of atypical self or reality monitoring, which is caused by the failure of the internal forward model (Cho and Wu, 2013; Moseley et al., 2013). Evidence from neuroimaging suggests that monitoring of one’s own speech, overt or covert, is related to activity in auditory cortical regions such as the left STG (Allen et al., 2007; Moseley et al., 2013). In addition, a greater CBF/FCS ratio in the left STG/MTG was positively linked with hallucination severity measured by AHRS in patients with AVHs, probably reflecting these regions based on trait study engaging in more rapid AVH processing. So, our findings of increased CBF/FCS in the left STG/MTG may implicate that the spontaneous auditory activation of auditory representation information emerged, the coordination and integration of that local activity across brain regions were impaired, and the event to another person was misattributed eventually.

In summary, we use the combination of BOLD and ASL technology to reveal the disordered coupling of resting CBF and functional connectivity between AVH and NAVH patients. In addition, our results revealed that schizophrenic patients had widespread deficits in both low-level sensorimotor and higher-order cognitive networks of the cerebellum, which suggest potential impairment affection, emotion, and cognitive functions. Specifically, our findings may possibly implicate that the typical symptom of AVHs in schizophrenia might arise from the failure of a forward model originating from functional synchronization abnormality among networks in cerebellar regions, which in turn might contribute to increase the activity of the cerebellum and alert the left STG/MTG by enhancing its excitability and, eventually, not recognizing that the experience is internally produced.

## Data Availability Statement

The original contributions presented in the study are included in the article/Supplementary Material, further inquiries can be directed to the corresponding author/s.

## Ethics Statement

This study has been reviewed and approved by the Ethics Committee of The First Affiliated Hospital of Zhengzhou University. Written informed consent to participate in this study was provided by the participants’ legal guardian/next of kin.

## Author Contributions

YW, JCheng, and SH designed the study and wrote the protocol. JChen performed the data processing and statistical analyses and wrote the first draft of the manuscript. All authors contributed to and have approved the final manuscript.

## Conflict of Interest

The authors declare that the research was conducted in the absence of any commercial or financial relationships that could be construed as a potential conflict of interest.

## Publisher’s Note

All claims expressed in this article are solely those of the authors and do not necessarily represent those of their affiliated organizations, or those of the publisher, the editors and the reviewers. Any product that may be evaluated in this article, or claim that may be made by its manufacturer, is not guaranteed or endorsed by the publisher.

## Footnotes

1
http://rfmri.org/dpabi

2
http://www.fil.ion.ucl.ac.uk/spm

3
www.humanbrain.cn

4
http://rfmri.org/
