# Supplementary material for: Altered Coupling of Cerebral Blood Flow and Functional Connectivity Strength in First-Episode Schizophrenia Patients With Auditory Verbal Hallucinations
Source: Front Neurosci. 2022 Apr 25;16:821078. doi: 10.3389/fnins.2022.821078 (PMC9083321; doi:10.3389/fnins.2022.821078)
Supplement: Supplementary file 1 [file Data_Sheet_1.docx]

Supplementary Material

# SupplementaryTables

**Table S1.Brain Regions withSignificant Group Differences in CBF between three groups**

| Group differences | Regions | Cluster size  (voxels) | Peak MNI coordinate | | | Peak T values |
| --- | --- | --- | --- | --- | --- | --- |
|  |  |  | X | Y | Z |  |
| *AVH/NAVH < HC* | R_ occipital middle gyrus | 248 | 26 | -96 | 6 | -12.76 |
|  | R_ occipital supper gyrus | 116 | -45 | -21 | 15 | -14.65 |
|  | R_ occipital inferior gyrus | 68 | 48 | -76 | -13 | -8.50 |
|  | R_ calcarine gyrus | 106 | 24 | -99 | 3 | -16.57 |
|  | R_ ligualgyrus | 28 | 11.51 | 9 | -93 | -6 |
| *AVH/NAVH > HC* | L_ putamen | 170 | -21 | 12 | 0 | 19.53 |
|  | L_ caudate | 40 | -30 | -3 | -12 | 12.08 |
| *NAVH < HC* | R_ cuneus | 71 | 13 | -91 | 23 | -13.13 |
| *AVH < HC* | L_ calcarinegyrus | 89 | -4 | -95 | -5 | -8.96 |
|  | L_ occipital middle gyrus | 65 | -21 | -99 | 3 | 11.59 |
|  | L_ occipital supper gyrus | 34 | -15 | -99 | 12 | 10.60 |
| *AVH> HC* | R_ putamen | 92 | 27 | 0 | 6 | 13.65 |
|  | R_ pallidum | 38 | 12 | -18 | 9 | 9.56 |
|  | R_ thalamus | 37 | 12 | -18 | 9 | 9.56 |
|  | L_ pallidum | 23 | -24 | -12 | 6 | 7.75 |
|  | R_ temporal inferior gyrus | 78 | 52 | -66 | -14 | 11.26 |
|  | R_ temporal middle gyrus | 59 | 51 | -66 | 3 | 9.53 |

Abbreviations: CBF, cerebral blood flow; AVH,schizophrenia patients with AVHs; NAVH, schizophrenia patients without AVHs; HC, healthy control; MNI, Montreal Neurological Institute. R, right; L, left.

**Table S2. Brain Regions with Significant Group Differences in FCS between three groups**

| Group differences | Regions | Cluster size  (voxels) | Peak MNI coordinate | | | Peak T values |
| --- | --- | --- | --- | --- | --- | --- |
|  |  |  | X | Y | Z |  |
| *AVH < HC* | R_inferior orbitofrontal gyrus | 39 | 28 | 25 | -23 | -9.25 |
|  | R_ supper temporal pole | 25 | 33 | 9 | -24 | -10.44 |
|  | R_parahippocampal | 48 | 24 | -5 | -33 | -9.58 |
|  | R_ fusiform | 22 | 30 | 0 | -36 | -7.49 |
| *NAVH> HC* | L_cerebebelum_crus Ⅱ | 122 | -3 | -84 | -27 | 14.68 |
|  | L_cerebellum_crus Ⅰ | 102 | -3 | -79 | -21 | 13.62 |
|  | vermis Ⅵ | 92 | 3 | -72 | -18 | 18.64 |
|  | L_cerebellum Ⅵ | 75 | -5 | -74 | -18 | 14.37 |
|  | L_cerebellum Ⅷ | 43 | -3 | -69 | -30 | 6.60 |
|  | vermis Ⅶ | 42 | 0 | -78 | -21 | 14.55 |
|  | R_cerebellum Ⅵ | 28 | 9 | -75 | -18 | 11.60 |

Abbreviations: FCS, functional connectivity strength; AVH, schizophrenia patients with AVHs; NAVH, schizophrenia patients without AVHs;HC, healthy control; MNI, Montreal Neurological Institute. R, right; L, left.

**Table S3. Correlations between CBF/FCS Ratio and Psychotic Symptoms^a^**

| Regions | PANSS positive | PANSS negative | | PANSS general | PANSS all |
| --- | --- | --- | --- | --- | --- |
| ***AVH < HC*** | | |  | | |
| L_cerebelum_crus Ⅰ  L_cerebelum_crus Ⅱ  R_cerebelum_crus Ⅱ  vermis Ⅵ  vermis Ⅶ | -0.167 (0.368)  -0.142 (0.445)  0.007 (0.971)  0.179 (0.335)  0.038 (0.841) | -0.075 (0.688)  -0.041 (0.828)  -0.160 (0.390)  0.089 (0.633)  -0.105 (0.576) | | -0.153 (0.410)  -0.037 (0.843)  -0.183 (0.324)  -0.036 (0.848)  -0.091 (0.628) | -0.204 (0.272)  -0.106 (0.570)  -0.125 (0.502)  0.117 (0.530)  -0.074 (0.690) |
| ***AVH > HC*** | | |  | | |
| L_MTG | -0.017 (0.929) | 0.070 (0.710) | | -0.088 (0.638) | -0.045 (0.812) |
| L_STG | 0.127 (0.497) | 0.011 (0.954) | | 0.087 (0.641) | 0.104 (0.579) |
| ***NAVH < HC*** |  |  | |  |  |
| L_cerebelum_Crus Ⅰ | -0.162 (0.323) | -0.106 (0.520) | | -0.106 (0.520) | -0.167 (0.311) |
| L_cerebelum_Crus Ⅱ | -0.052 (0.756) | -0.180 (0.273) | | -0.065 (0.694) | -0.142 (0.389) |
| vermis Ⅵ | -0.114 (0.489) | -0.114 (0.489) | | -0.088 (0.594) | -0.121 (0.463) |
| vermis Ⅶ | -0.071 (0.668) | -0.139 (0.398) | | -0.108 (0.514) | -0.145 (0.379) |

Abbreviations: CBF, cerebral blood flow; FCS, functional connectivity strength.

^a^The data are shown as the Spearman’s rho (*P* value); PANSS,the Positive and Negative Symptom Scale; L_MTG,left middle temporal gyrus; L_STG, left superior gyrus. R, right; L, left. AVH, schizophrenia patients with AVHs; NAVH, schizophrenia patients without AVHs; HC, healthy control;

**Table S4. Correlations between CBF/FCS Ratio and** **AHRS ^a^**

| Regions | Hoffman all |
| --- | --- |
| *AVH <HC* |  |
| L_cerebelum_Crus Ⅰ | -0.078 (0.608) |
| L_cerebelum_Crus Ⅱ | -0.114 (0.449) |
| R_cerebelum_Crus Ⅱ | -0.074 (0.627) |
| Vermis Ⅵ | -0.030 (0.845) |
| Vermis Ⅶ | -0.047 (0.754) |
| *AVH >HC* |  |
| L_MTG | **0.343 (0.020)*** |
| L_STG | **0.303 (0.041)*** |

Abbreviations: CBF, cerebral blood flow; FCS, functional connectivity strength.AHRS:Auditory Hallucination Rating Scale; ^a^ The data are shown as the Spearman’s *rho* (*P* value); *****Significant for *P*< 0.05; L_MTG,left middle temporal gyrus; L_STG, left superior gyrus. R, right; L, left. AVH,schizophrenia patients with AVHs; NAVH, schizophrenia patients without AVHs; HC, healthy control;

**Table S5. Brain Regions with Significant Group Differences in CBF/FCS ratio**

| Group differences | Regions | Cluster size  (voxels) | Peak MNI coordinate | | | Peak T values |
| --- | --- | --- | --- | --- | --- | --- |
|  |  |  | X | Y | Z |  |
| *AVH> HC* | L_STG | 106 | -54 | -30 | 9 | 9.32 |
|  | L_MTG | 35 | -69 | -27 | -3 | 7.77 |
| *AVH/ NAVH < HC* | Vermis Ⅵ | 72 | 3 | -72 | -18 | -18.78 |
|  | L_cerebellum crus Ⅱ | 58 | -3 | -87 | -27 | -14.75 |
|  | L_cerebellum Ⅵ | 50 | -6 | -72 | -21 | -9.50 |
|  | L_cerebellum crus Ⅰ | 46 | -33 | -90 | -30 | -8.22 |
|  | vermis Ⅶ | 31 | 3 | -72 | -24 | -10.68 |
| *AVH < HC* | R_cerebellum crus Ⅱ | 23 | 6 | -87 | -30 | -10.32 |

Abbreviations:AVH, schizophrenia with AVHs patients; NAVH, schizophrenia patients without AVHs; HC, healthy control; MNI, Montreal Neurological Institute; L_MTG, left middle temporal gyrus; L_STG, left superior temporal gyrus;

**Table S6. Brain Regions with Significant Group Differences in CBF/FCS ratio**

| Group differences | Regions | Cluster size  (voxels) | Peak MNI coordinate | | | Peak T values |
| --- | --- | --- | --- | --- | --- | --- |
|  |  |  | X | Y | Z |  |
|  | L_STG | 89 | -54 | -30 | 9 | 9.32 |
|  | L_MTG | 34 | -69 | -27 | -3 | 7.77 |
| *AVH/ NAVH < HC* | vermisⅥ | 71 | 3 | -72 | -18 | -18.91 |
|  | L_cerebellum crus Ⅱ | 56 | -3 | -87 | -27 | -15.12 |
|  | L_cerebellum Ⅵ | 51 | -6 | -72 | -21 | -9.50 |
|  | L_cerebellum crus Ⅰ | 53 | -33 | -90 | -30 | -8.34 |
|  | vermis Ⅶ | 26 | 3 | -72 | -24 | -10.68 |
| *AVH < HC* | R_cerebellum crus Ⅱ | 22 | 6 | -87 | -30 | -10.32 |

Abbreviations:AVH, schizophrenia with AVHs patients; NAVH, schizophrenia without AVHs patients; HC, healthy control; MNI, Montreal Neurological Institute; L_MTG, left middle temporal gyrus; L_STG, left superior temporal gyrus;

# Supplementary Figures


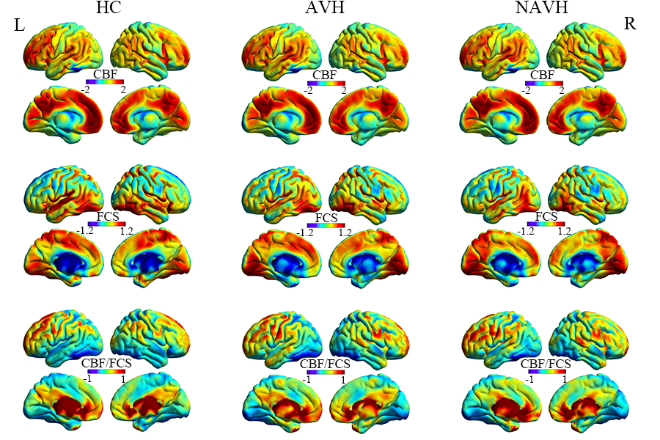


**Supplementary FigureS1.**Spatial distribution maps of FCS, CBF, and CBF/FCS ratio. The CBF, FCS, and CBF/FCS ratio maps were averaged across subjects within groups. CBF, cerebral blood flow; FCS, functional connectivity strength; HC, healthy controls; AVH, schizophrenia with AVH patients; NAVH, schizophrenia without AVH patients;L, left; R, right.


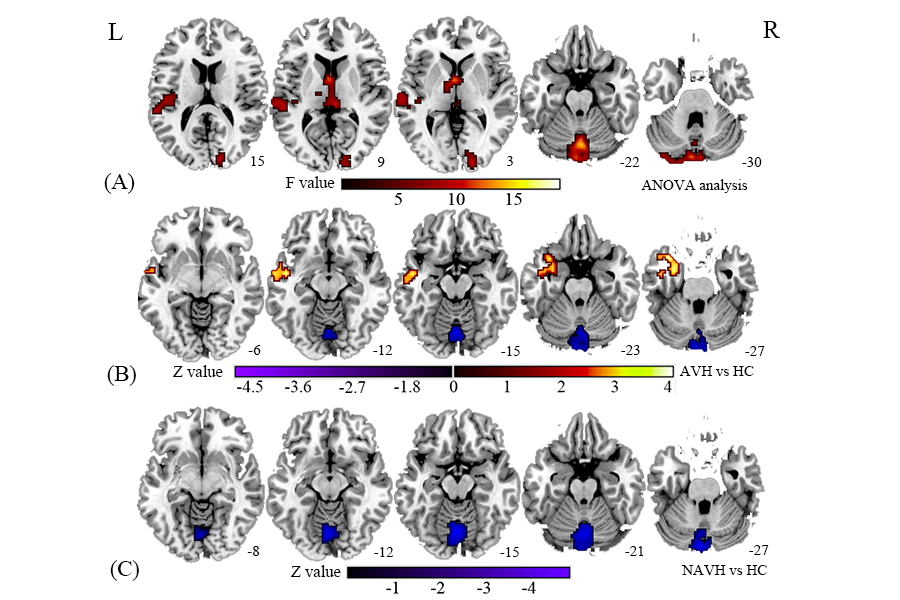


**Supplementary FigureS2**(A) CBF/FCS ratio (at the connectivity threshold of 0.1) results for HC, AVH, NAVH groups at a voxel-level height threshold of p < 0.005 (2-sided) and cluster size GRF corrected threshold of P <0.05. (B) Brain regions with significant CBF/FCS changes in the AVH group. (C) Brain regions with significant CBF/FCS changes in the NAVH group. CBF, cerebral blood flow; FCS, functional connectivity strength.


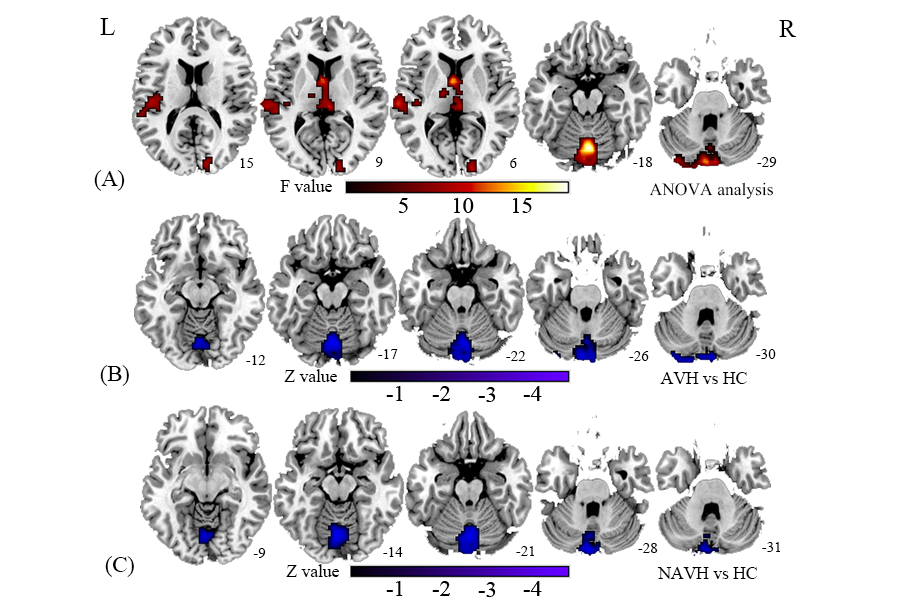


**Supplementary FigureS3** (A) CBF/FCS ratio (at the connectivity threshold of 0.3) results for HC, AVH, NAVH groups at a voxel-level height threshold of p < 0.005 (2-sided) and cluster size GRF corrected threshold of P <0.05. (B)Brain regions with significant CBF/FCS changes in the AVH group. (C)Brain regions with significant CBF/FCS changes in the NAVH group. CBF, cerebral blood flow; FCS, functional connectivity strength.


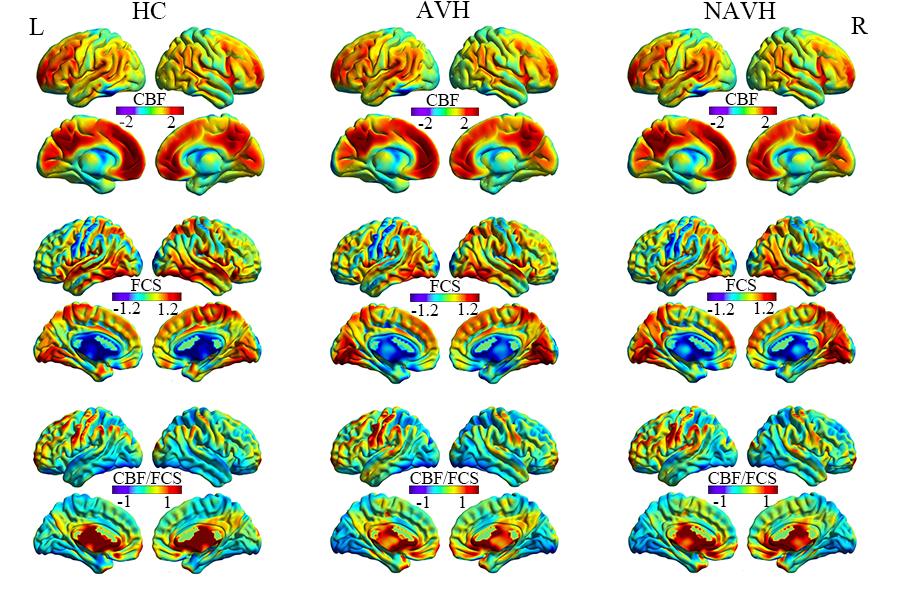


**Supplementary FigureS4.**Spatial distribution maps of FCS (at the threshold of 0.1), CBF and CBF/FCS ratio.The CBF, FCS, and CBF/FCS ratio maps were averaged across subjects within groups. CBF,cerebral blood flow; FCS, functional connectivity strength; HC, healthy controls; AVH, schizophrenia with AVHs patients; NAVH, schizophrenia without AVHs patients; L, left; R, right;


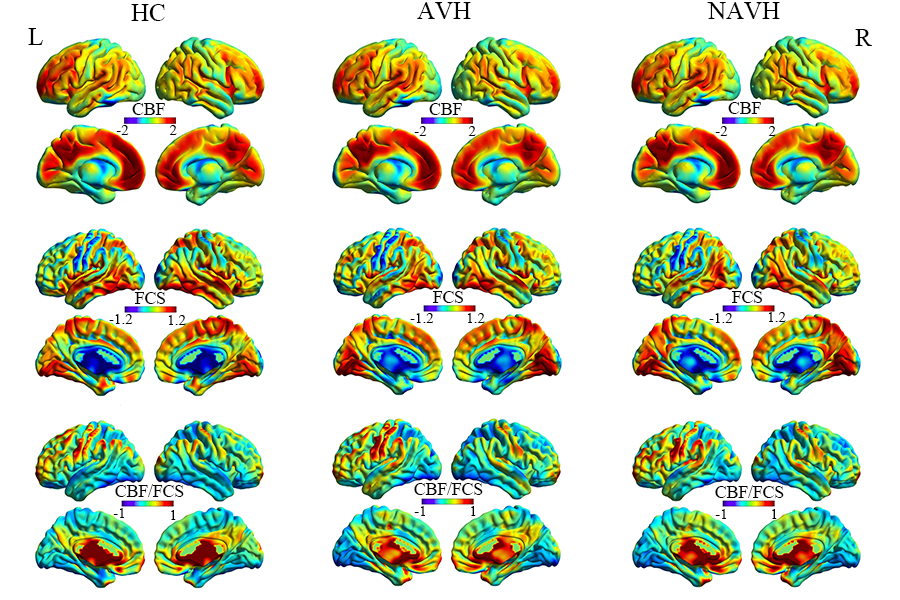


**Supplementary FigureS5.**Spatial distribution maps of FCS (at the threshold of 0.3), CBF and CBF/FCS ratio.The CBF, FCS, and CBF/FCS ratio maps were averaged across subjects within groups. CBF,cerebral blood flow; FCS, functional connectivity strength; HC, healthy controls; AVH, schizophrenia with AVHs patients; NAVH, schizophrenia without AVHs patients; L, left; R, right;


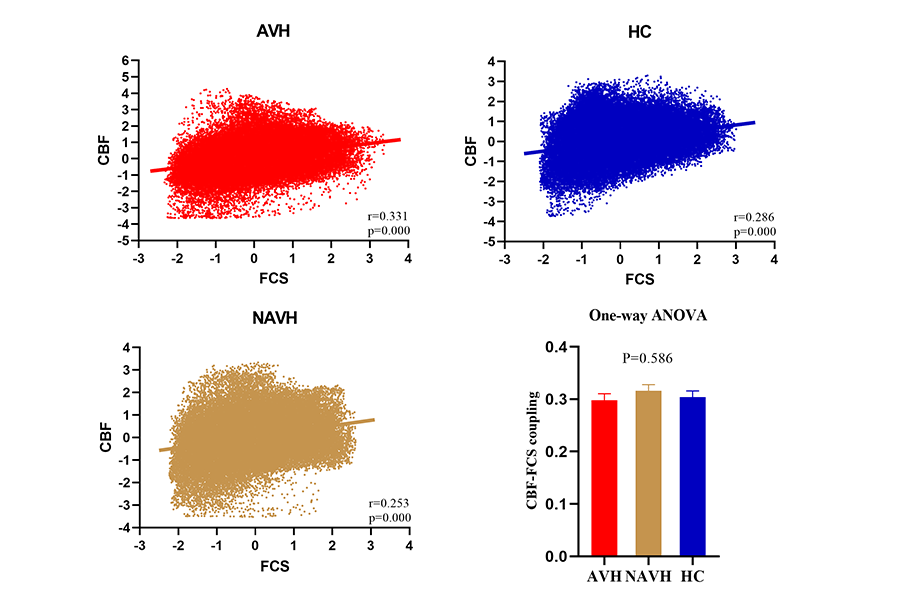


**Figure S6.**: Whole gray matter level CBF-FCS coupling changes in schizophrenia(at the threshold of 0.1). Scatter plots of the spatial correlations across voxels between CBF and FCS in a AVH (red), a NAVH patient (yellow), and a HC subject (blue), respectively. The mean whole gray matter level CBF-FCS coupling in AVH patients, NAVH patients, and HC. Although CBF is significantly correlated with FCS in both schizophrenia and control groups, there was no difference between the three groups. CBF, cerebral blood flow; FCS, functional connectivity strength; AVH, schizophrenia patients with AVHs; NAVH, schizophrenia patients without AVHs; HC, healthy control.


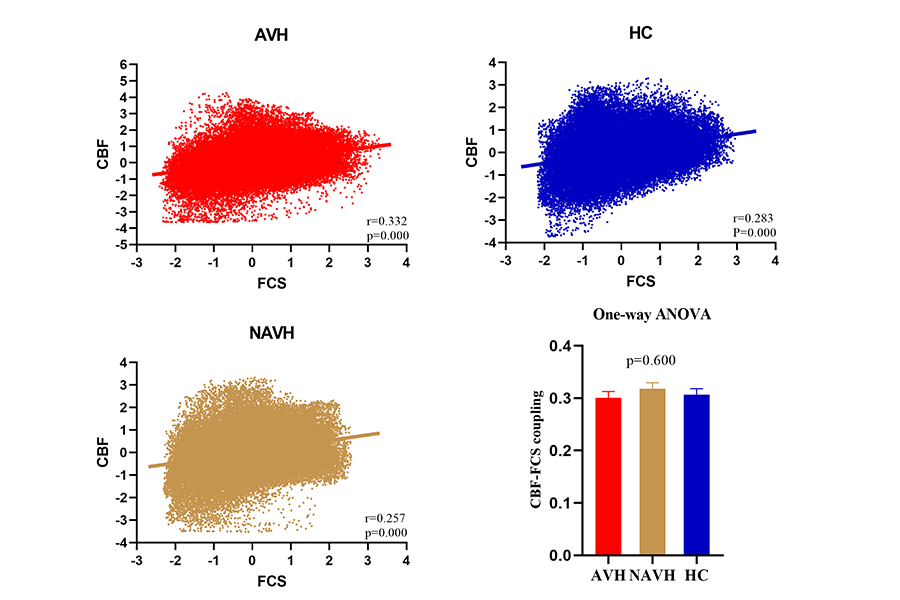


**Figure S7.**: Whole gray matter level CBF-FCS coupling changes in schizophrenia(at the threshold of 0.3). Scatter plots of the spatial correlations across voxels between CBF and FCS in a AVH (red), a NAVH patient (yellow), and a HC subject (blue), respectively. The mean whole gray matter level CBF-FCS coupling in AVH patients, NAVH patients, and HC. Although CBF is significantly correlated with FCS in both schizophrenia and control groups, there was no difference between the three groups. CBF, cerebral blood flow; FCS, functional connectivity strength; AVH, schizophrenia patients with AVHs; NAVH, schizophrenia patients without AVHs; HC, healthy control.


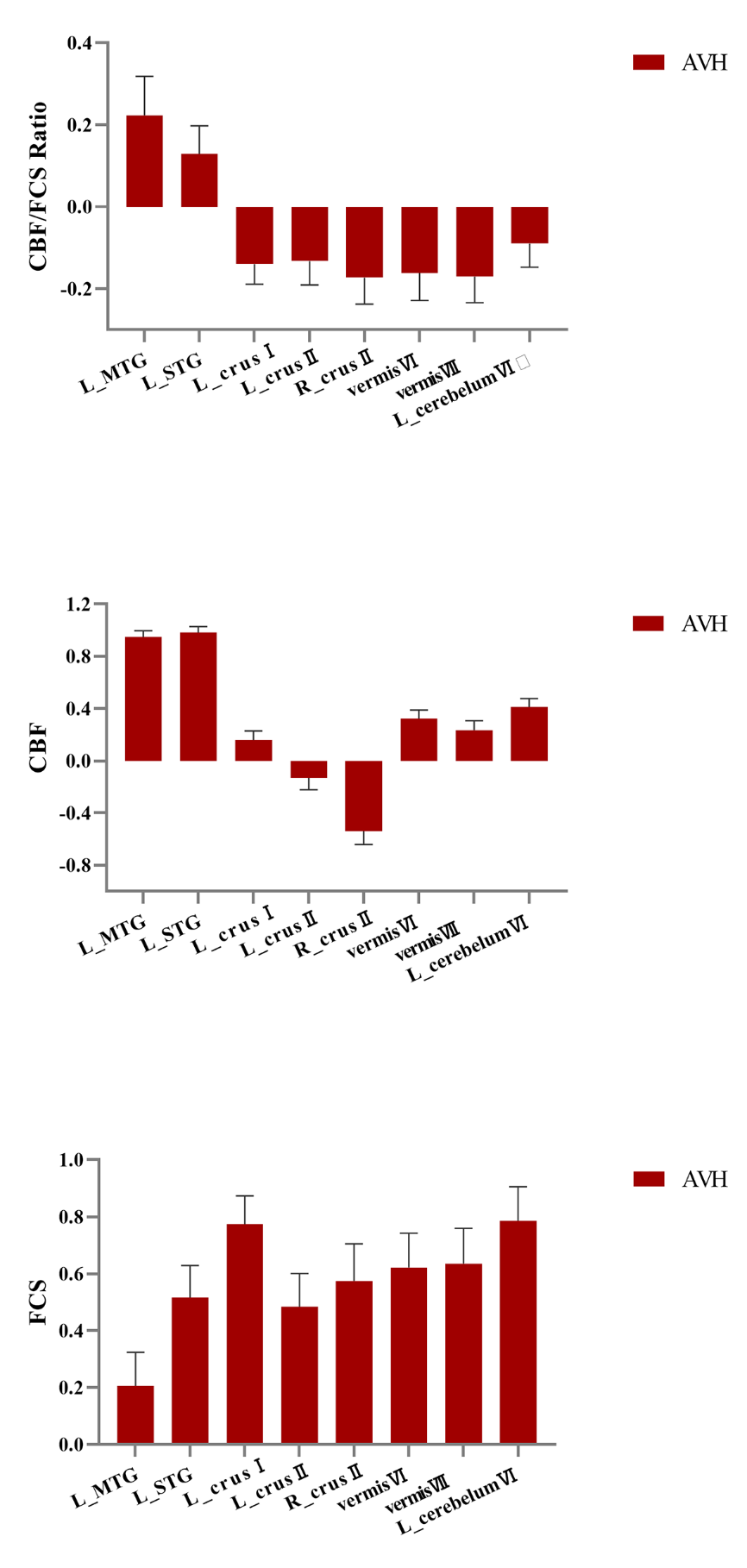


**Supplementary FigureS8.**ROI-based analyses for brain regions exhibiting no significant intergroup differences in CBF and FCS but significant differences in CBF/FCS ratio in the voxel-based analyses. Error bars indicate the standard error of the mean. Abbreviations: ROI, region of interest; CBF, cerebral blood flow; FCS, functional connectivity strength; AVH, first-episode drug-naïve schizophrenia (FES) patients with AVHs.
